# Survival after pathological complete response following neoadjuvant chemotherapy *versus* chemoradiotherapy for oesophageal squamous cell carcinoma

**DOI:** 10.1093/bjs/znag012

**Published:** 2026-02-17

**Authors:** Jun Okui, Satoru Matsuda, Kengo Nagashima, Yasunori Sato, Hirofumi Kawakubo, Thomas Ruhstaller, Peter Thuss-Patience, Magnus Nilsson, Fredrik Klevebro, Lijie Tan, Shaoyuan Zhang, Thomas Aparicio, Guillaume Piessen, Charlène van der Zijden, Bianca Mostert, Bas P L Wijnhoven, Takahiro Tsushima, Hiroya Takeuchi, Ken Kato, Yuko Kitagawa

**Affiliations:** Department of Surgery, Keio University School of Medicine, Tokyo, Japan; Department of Biostatistics, Keio University School of Medicine, Tokyo, Japan; Department of Surgery, Keio University School of Medicine, Tokyo, Japan; Biostatistics Unit, Clinical and Translational Research Center, Keio University Hospital, Tokyo, Japan; Department of Biostatistics, Keio University School of Medicine, Tokyo, Japan; Department of Surgery, Keio University School of Medicine, Tokyo, Japan; Competence Center, Swiss Cancer Institute, Bern, Switzerland; Faculty of Medicine, University of Basel, Basel, Switzerland; Department of Haematology, Oncology, and Cancer Immunology, Charité-University Medicine Berlin, Campus Virchow Klinikum, Berlin, Germany; Department of Upper Abdominal Diseases, Karolinska University Hospital, Stockholm, Sweden; Division of Surgery and Oncology, Department of Clinical Science, Intervention, and Technology (CLINTEC), Karolinska Institutet, Stockholm, Sweden; Department of Upper Abdominal Diseases, Karolinska University Hospital, Stockholm, Sweden; Division of Surgery and Oncology, Department of Clinical Science, Intervention, and Technology (CLINTEC), Karolinska Institutet, Stockholm, Sweden; Department of Thoracic Surgery, Zhongshan Hospital, Fudan University, Shanghai, China; Department of Thoracic Surgery, Zhongshan Hospital, Fudan University, Shanghai, China; Department of Digestive Oncology, Hôpital Saint-Louis, APHP, Université Paris Cité, Paris, France; UMR9020-U1277—CANTHER—Cancer Heterogeneity, Plasticity, and Resistance to Therapies, University Lille, CNRS, Inserm, CHU Lille, Lille, France; Department of Surgery, Erasmus MC Cancer Institute, Erasmus University Medical Centre, Rotterdam, The Netherlands; Department of Medical Oncology, Erasmus MC Cancer Institute, Erasmus University Medical Centre, Rotterdam, The Netherlands; Department of Surgery, Erasmus MC Cancer Institute, Erasmus University Medical Centre, Rotterdam, The Netherlands; Division of Gastrointestinal Oncology, Shizuoka Cancer Center, Shizuoka, Japan; Department of Surgery, Hamamatsu University School of Medicine, Shizuoka, Japan; Department of Head and Neck, Oesophageal Medical Oncology, National Cancer Center Hospital, Tokyo, Japan; Department of Surgery, Keio University School of Medicine, Tokyo, Japan

## Abstract

**Background:**

Although several oesophageal squamous cell carcinoma (OSCC) studies have reported no definitive overall survival (OS) differences between neoadjuvant chemoradiotherapy (NACRT) and neoadjuvant chemotherapy (NAC), the higher pCR rate with NACRT has been viewed as a potential advantage. Beyond ongoing concerns about the validity of pCR as a surrogate endpoint, it remains uncertain whether survival differs between these modalities among patients with OSCC who achieve pCR.

**Methods:**

An integrated analysis of individual patient data (IPD) from phase III trials evaluating perioperative therapies for resectable OSCC was conducted, emphasizing prognostic differences between NAC and NACRT, particularly among patients who achieved pCR.

**Results:**

IPD from seven phase III RCTs across six countries included data for 1044 patients with OSCC (83.5% male; mean age of 62.3 years). Of these patients, 605 (58.0%) received NAC and 439 (42.0%) received NACRT, with R0 resection rates of 89.6% *versus* 84.7% and pCR rates of 6.9% *versus* 34.2% respectively. Among patients who achieved pCR (192 patients), 5-year OS was 97.5% in the NAC group and 70.4% in the NACRT group, while 5-year recurrence-free survival was 80.8% and 63.7% respectively. Multivariable analysis demonstrated a significant survival advantage for NAC among patients who achieved pCR.

**Conclusion:**

Among patients who achieved pCR, postoperative outcomes varied considerably by neoadjuvant treatment modality. The markedly favourable prognosis associated with pCR after NAC suggests that these patients may represent an optimal candidate cohort for future evaluation of surgery-avoidance and watch-and-wait strategies.

## Teaser

In an integrated analysis of individual patient data from seven phase III randomized trials of resectable oesophageal squamous cell carcinoma, survival outcomes among patients achieving pCR differed substantially by neoadjuvant treatment modality. Despite a higher pCR rate with neoadjuvant chemoradiotherapy, patients achieving pCR after neoadjuvant chemotherapy had significantly superior long-term overall and recurrence-free survival. These findings indicate that pCR is not an equivalent prognostic endpoint across treatment modalities and suggest that pCR after chemotherapy may identify patients suitable for organ-preserving strategies.

## Introduction

Globally, oesophageal cancer ranks 11th with regard to cancer incidence and 7th with regard to cancer-related mortality^1^. Because oesophageal cancer can spread from the neck to the abdomen, even in early stages, leading to systemic progression^2–4^, multidisciplinary treatment that includes surgery, chemotherapy, radiotherapy, and immunotherapy is required^5–7^. In Japan, neoadjuvant chemotherapy (NAC) followed by oesophagectomy with radical lymph node dissection has shown benefit, particularly for oesophageal squamous cell carcinoma (OSCC)^8^. In Western countries, standard therapy for surgically resectable OSCC either involves neoadjuvant chemoradiotherapy (NACRT) or perioperative chemotherapy with transthoracic oesophagectomy^9–11^. Trials such as ESOPEC, NeoRes, and Neo-AEGIS have not demonstrated survival differences between NACRT and NAC for patients with oesophageal adenocarcinoma; likewise, the JCOG1109 and CMISG1701 trials showed no survival advantage of NACRT over NAC for OSCC^12–16^. Although NACRT results in higher pCR rates and greater tumour regression, these benefits have not translated into improved survival.

Several explanations have been proposed for this observed discrepancy in response compared with survival. In JCOG1109, patients receiving NAC were more likely to undergo diverse post-recurrence treatments, including chemoradiotherapy or radiotherapy, whereas the NACRT cohort had more non-cancer-related deaths, both of which may have influenced the observed outcomes^14^. In addition, previous work demonstrated that pCR lacks individual-level surrogacy for overall survival (OS), suggesting that pCR may be unsuitable as a primary endpoint in phase III randomized trials^17^. Supporting this concern, Cools-Lartigue *et al*.^18^ reported that, among pCR (ypT0N0) patients with oesophageal adenocarcinoma, survival differed between those receiving NAC and those receiving NACRT, further challenging pCR as a surrogate endpoint. However, beyond concerns regarding the validity of pCR as a surrogate for OS, it remains unclear whether survival differs between NAC and NACRT among patients with OSCC who achieve pCR.

Therefore, the aim of this study was to evaluate whether postoperative survival differs between NAC and NACRT among patients with OSCC who achieve pCR using individual patient data (IPD) from phase III randomized trials of resectable disease. The central hypothesis was that patients receiving NAC would demonstrate more favourable post-pCR survival than those receiving NACRT.

## Methods

### Study design

This study involved an integrated analysis of IPD from RCTs that compared perioperative therapies for surgically resectable advanced oesophageal and gastro-oesophageal junction cancer (International Collaborative Research Project, ‘JP-ESOP23’)^17,19,20^. This study was approved by the Keio University School of Medicine Ethics Committee (approval number: 2023-1032). The study was performed in accordance with the Declaration of Helsinki. The requirement for informed consent was waived because of the retrospective study design. This study adhered to the STROBE guidelines, given that it was designed as a retrospective cohort study based on available IPD^21^.

A systematic review was initially conducted and requests for IPD were sent to the principal investigators of all eligible trials. The review was registered in PROSPERO, the international prospective register of systematic reviews (registration number: CRD42023396321), with additional details reported elsewhere^17,19,20^. Trials were considered eligible if they were phase III RCTs evaluating perioperative therapies for resectable oesophageal or gastro-oesophageal junction cancer and had closed to patient accrual before 31 December 2020. Trials that compared perioperative therapies with immune checkpoint inhibitors (ICIs) were excluded to enhance internal validity, as only a few phase III trials for resectable oesophageal cancer were available or had published results at the time of planning.

### Patient selection

Inclusion criteria for patients were: age ≥18 years; diagnosis of thoracic oesophageal cancer or gastro-oesophageal junction cancer; histological confirmation of squamous cell carcinoma; clinical stage I–III disease, excluding cT1N0 and cT4b, based on the eighth edition of the UICC TNM classification^22^; and assignment to an NAC or NACRT arm. Patients were excluded if they opted out, if OS data were unavailable, or if they were assigned to treatment arms that included molecular-targeted agents.

### Outcomes

All clinicopathological data were collected from the IPD of each trial and reanalysed centrally and checked for inconsistencies. The primary endpoint was OS and the secondary endpoints included R0 resection rate, pathological response, recurrence-free survival (RFS), and cause of death (cancer-related or non-cancer-related). OS was calculated from the date of randomization to the date of death or last follow-up. RFS was calculated from the date of randomization to the date of death, recurrence, or last follow-up^23^. In the overall cohort analyses, pathological response was defined using pathological grade (pGrade)^24,25^ or Mandard tumour regression grade (TRG)^26^, depending on the trial. pCR was defined as ypT0N0.

### Statistical analysis

All analyses were performed using R software, version 4.5.1 (R Foundation for Statistical Computing, Vienna, Austria).

The Kaplan–Meier method was used to estimate OS and RFS, with emphasis on 5-year survival rates and median survival times. Comparisons between the NAC and NACRT groups were performed using the log rank test. Multivariable Cox proportional hazard models were used to evaluate survival-time endpoints, with covariates that included sex, age at randomization, cT category, cN category, and preoperative treatment arm (NAC *versus* NACRT). HRs and 95% confidence intervals were also estimated. Analyses were conducted for patients who achieved pCR and repeated for the non-pCR cohort as exploratory analyses. Continuous variables are reported as mean (s.d.) and were analysed using Welch’s *t* test, whereas categorical variables are reported as *n* (%) and were analysed using the chi-squared test. All *P* values are two-tailed and *P* < 0.050 was considered statistically significant. Missing values were not imputed.

## Results

The literature search, study selection, and IPD selection process are presented in *Fig. S1*. Of the 22 eligible trials, 10 provided IPD across six countries (*Table S1*)^8,11,14,15,27–32^. Among these trials, the JCOG9204 trial was excluded because neither NAC nor NACRT was included in its treatment arms^30^. The Boonstra *et al*.^27^ and FFCD9102^28^ trials were also excluded because their treatment approaches were insufficiently contemporary, given major changes in therapeutic standards. One arm of the SAKK75/08 trial that incorporated a molecular-targeted agent was also excluded to maintain focus on chemotherapy-based comparisons^32^. For the CMISG1701 trial, IPD were only available for patients enrolled at certain facilities^15^. Overall, 1044 patients with OSCC (clinical stages I–III, excluding cT1N0 and cT4b) were included (*Fig. S1*).

Baseline characteristics for the 1044 patients are summarized in *Table 1*. The mean age was 62.3 years and 83.5% were male. Of the full cohort, 605 patients (58.0%) received NAC and 439 patients (42.0%) received NACRT. The two groups showed no major differences in baseline characteristics, including age, sex, and clinical stage. The R0 resection rate was 89.6% (542 of 605) in the NAC group and 84.7% (372 of 439) in the NACRT group. Tumour response in the primary lesion differed considerably: the pCR rate was 6.9% (42 of 605) in the NAC group and 34.2% (150 of 439) in the NACRT group. pGrade- and TRG-based assessments similarly indicated more favourable tumour regression with NACRT compared with NAC (*Table 1*).

**Table 1:** Patient characteristics

	Overall (*n* = 1044)	NAC (*n* = 605)	NACRT (*n* = 439)
**Trial**			
CMISG1701	80 (7.7)	41 (6.8)	39 (8.9)
CROSS	41 (3.9)	0 (0.0)	41 (9.3)
FFCD9901	55 (5.3)	0 (0.0)	55 (12.5)
JCOG1109	598 (57.3)	400 (66.1)	198 (45.1)
JCOG9907	164 (15.7)	164 (27.1)	0 (0.0)
NeoRes2	51 (4.9)	0 (0.0)	51 (11.6)
SAKK75/08	55 (5.3)	0 (0.0)	55 (12.5)
**NAC regimen**			
Cisplatin and 5-FU	616 (59.0)	363 (60.0)	253 (57.6)
Docetaxel, cisplatin, and 5-FU	201 (19.3)	201 (33.2)	0 (0.0)
Paclitaxel and carboplatin	92 (8.8)	0 (0.0)	92 (21.0)
Paclitaxel and cisplatin	80 (7.7)	41 (6.8)	39 (8.9)
Docetaxel and cisplatin	55 (5.3)	0 (0.0)	55 (12.5)
Age (years), mean(s.d.)	62.3(7.8)	62.7(7.5)	61.9(8.3)
**Sex**			
Male	872 (83.5)	534 (88.3)	338 (77.0)
Female	172 (16.5)	71 (11.7)	101 (23.0)
**PS**			
0	837 (80.4)	489 (81.0)	348 (79.6)
1	204 (19.6)	115 (19.0)	89 (20.4)
**Tumour location**			
Oesophageal cancer	1032 (98.9)	605 (100.0)	427 (97.5)
Gastro-oesophageal junction cancer	11 (1.1)	0 (0.0)	11 (2.5)
**cT**			
1	72 (7.3)	49 (8.1)	23 (6.0)
2	190 (19.2)	118 (19.5)	72 (18.8)
3	697 (70.5)	431 (71.2)	266 (69.5)
4a	29 (2.9)	7 (1.2)	22 (5.7)
**cN**			
0	256 (24.6)	150 (24.8)	106 (24.3)
1	580 (55.7)	331 (54.7)	249 (57.0)
2	185 (17.8)	116 (19.2)	69 (15.8)
3	18 (1.7)	8 (1.3)	10 (2.3)
cN+	3 (0.3)	0 (0.0)	3 (0.7)
**cStage**			
I	53 (5.2)	33 (5.5)	20 (4.8)
II	373 (36.7)	211 (35.3)	162 (38.8)
III	589 (58.0)	354 (59.2)	235 (56.4)
**ypT**			
0	232 (24.7)	48 (8.6)	184 (47.7)
is	8 (0.9)	8 (1.4)	0 (0.0)
1	182 (19.3)	120 (21.6)	62 (16.1)
2	132 (14.0)	77 (13.9)	55 (14.2)
3	363 (38.6)	283 (51.0)	80 (20.7)
4	24 (2.6)	19 (3.4)	5 (1.3)
**ypN**			
0	479 (50.8)	222 (39.7)	257 (67.1)
1	312 (33.1)	235 (42.0)	77 (20.1)
2	99 (10.5)	74 (13.2)	25 (6.5)
3	39 (4.1)	28 (5.0)	11 (2.9)
ypN+	13 (1.4)	0 (0.0)	13 (3.4)
ypM1	64 (6.6)	52 (9.3)	12 (2.9)
**ypStage**			
I	363 (38.7)	139 (25.0)	224 (58.5)
II	100 (10.7)	72 (13.0)	28 (7.3)
III	365 (38.9)	259 (46.7)	106 (27.7)
IV	110 (11.7)	85 (15.3)	25 (6.5)
**Resection margin status**			
No surgery	98 (9.4)	41 (6.8)	57 (13.0)
R0	914 (87.5)	542 (89.6)	372 (84.7)
R1	10 (1.0)	5 (0.8)	5 (1.1)
R2	22 (2.1)	17 (2.8)	5 (1.1)
pCR (ypT0N0)	192 (18.4)	42 (6.9)	150 (34.2)
**pGrade**			
0	50 (7.3)	47 (9.2)	3 (1.7)
1a	247 (36.0)	232 (45.5)	15 (8.5)
1b	86 (12.5)	65 (12.7)	21 (11.9)
2	173 (25.2)	113 (22.2)	60 (33.9)
3	131 (19.1)	53 (10.4)	78 (44.1)
**TRG**			
1	96 (42.7)	2 (5.4)	94 (50.0)
2	47 (20.9)	1 (2.7)	46 (24.5)
3	26 (11.6)	8 (21.6)	18 (9.6)
4	56 (24.9)	26 (70.3)	30 (16.0)

Values are *n* (%) unless otherwise indicated. NAC, neoadjuvant chemotherapy; NACRT, neoadjuvant chemoradiotherapy; 5-FU, 5-fluorouracil; PS, performance status; cStage, clinical stage; ypStage, post-neoadjuvant pathologic stage; pGrade, pathological grade; TRG, tumour regression grade.

**Fig. 1:**
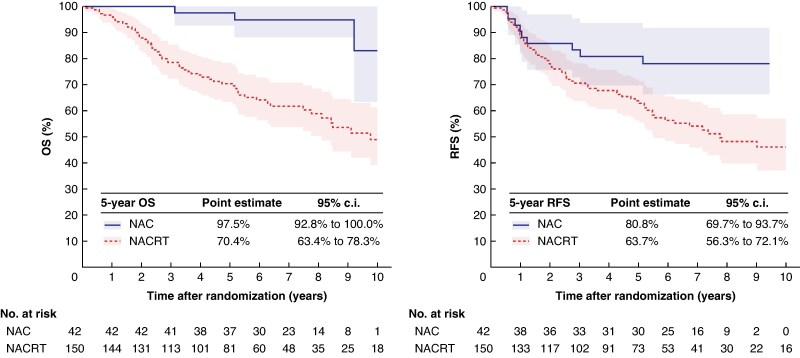
Kaplan–Meier estimates of OS and RFS among patients who achieved pCR Shaded areas represent 95% confidence intervals. OS, overall survival; RFS, recurrence-free survival; NAC, neoadjuvant chemotherapy; NACRT, neoadjuvant chemoradiotherapy.

The baseline characteristics of the patients who achieved pCR (192 patients) are summarized in *Table 2*; for these patients, survival outcomes differed markedly between treatment groups (*Fig. 1*). The 5-year OS was 97.5% (95% c.i. 92.8% to 100.0%) in the NAC group and 70.4% (95% c.i. 63.4% to 78.3%) in the NACRT group. The 5-year RFS was 80.8% (95% c.i. 69.7% to 93.7%) and 63.7% (95% c.i. 56.3% to 72.1%) respectively. Median OS was not reached in the NAC group and was 9.8 (95% c.i. 8.1 to not estimable) years in the NACRT group. Median RFS was not reached in the NAC group and was 7.8 (95% c.i. 5.5 to not estimable) years in the NACRT group. Differences in OS and RFS were statistically significant (OS: *P* < 0.001; RFS: *P* = 0.01; *Fig. 1*). In contrast, survival differences were smaller in the non-pCR cohort (748 patients; patients for whom pCR could not be assessed, such as those who did not undergo surgical resection, were excluded). The 5-year OS was 57.4% (95% c.i. 53.2% to 61.9%) in the NAC group and 47.8% (95% c.i. 41.6% to 54.8%) in the NACRT group. The 5-year RFS was 48.0% (95% c.i. 43.8% to 52.6%) and 40.6% (95% c.i. 34.6% to 47.6%) respectively (*Fig. S2*). Additionally, among the 42 patients who achieved pCR after NAC, 7 experienced recurrence and 3 died; all of the deaths were due to non-cancer-related causes (100%). In contrast, among the 150 patients who achieved pCR after NACRT, 36 experienced recurrence and 59 died. The cause of death was unknown for 14 patients. Among the remaining 45 patients with available data regarding the cause of death, 24 (53.3%) and 21 (46.7%) died from cancer-related and non-cancer-related causes respectively.

**Table 2:** Baseline characteristics of patients who achieved pCR

	Overall (*n* = 192)	NAC (*n* = 42)	NACRT (*n* = 150)
**Trial**			
CMISG1701	15 (7.8)	2 (4.8)	13 (8.7)
CROSS	19 (9.9)	0 (0.0)	19 (12.7)
FFCD9901	16 (8.3)	0 (0.0)	16 (10.7)
JCOG1109	103 (53.6)	36 (85.7)	67 (44.7)
JCOG9907	4 (2.1)	4 (9.5)	0 (0.0)
NeoRes2	18 (9.4)	0 (0.0)	18 (12.0)
SAKK75/08	17 (8.9)	0 (0.0)	17 (11.3)
**NAC regimen**			
Cisplatin and 5-FU	91 (47.4)	8 (19.0)	83 (55.3)
Docetaxel, cisplatin, and 5-FU	32 (16.7)	32 (76.2)	0 (0.0)
Paclitaxel and carboplatin	37 (19.3)	0 (0.0)	37 (24.7)
Paclitaxel and cisplatin	15 (7.8)	2 (4.8)	13 (8.7)
Docetaxel and cisplatin	17 (8.9)	0 (0.0)	17 (11.3)
Age (years), mean(s.d.)	62.5(8.5)	65.1(8.0)	61.7(8.5)
**Sex**			
Male	154 (80.2)	37 (88.1)	117 (78.0)
Female	38 (19.8)	5 (11.9)	33 (22.0)
**PS**			
0	160 (83.8)	39 (92.9)	121 (81.2)
1	31 (16.2)	3 (7.1)	28 (18.8)
**Tumour location**			
Oesophageal cancer	187 (97.4)	42 (100.0)	145 (96.7)
Gastro-oesophageal junction cancer	5 (2.6)	0 (0.0)	5 (3.3)
**cT**			
1	16 (9.1)	6 (14.3)	10 (7.5)
2	40 (22.7)	15 (35.7)	25 (18.7)
3	113 (64.2)	21 (50.0)	92 (68.7)
4a	7 (4.0)	0 (0.0)	7 (5.2)
**cN**			
0	48 (25.0)	11 (26.2)	37 (24.7)
1	110 (57.3)	21 (50.0)	89 (59.3)
2	30 (15.6)	9 (21.4)	21 (14.0)
3	3 (1.6)	1 (2.4)	2 (1.3)
cN+	1 (0.5)	0 (0.0)	1 (0.7)
**cStage**			
I	17 (9.1)	8 (19.0)	9 (6.2)
II	68 (36.6)	14 (33.3)	54 (37.5)
III	101 (54.3)	20 (47.6)	81 (56.2)

Values are *n* (%) unless otherwise indicated. NAC, neoadjuvant chemotherapy; NACRT, neoadjuvant chemoradiotherapy; 5-FU, 5-fluorouracil; PS, performance status; cStage, clinical stage.

In the pCR cohort, multivariable Cox proportional hazards models adjusting for sex, age at randomization, cT category, cN category, and treatment arm showed that NAC, compared with NACRT, was independently associated with superior OS and RFS (OS: HR 0.12 (95% c.i. 0.04 to 0.41), *P* = 0.001; RFS: HR 0.40 (95% c.i. 0.20 to 0.84), *P* = 0.015; *Table 3*).

**Table 3:** Multivariable Cox regression analyses for OS and RFS among patients with pCR

Outcome	Variable		HR (95% c.i.)	*P*
OS	Age	Continuous	1.04 (1.00,1.08)	0.040
	Sex	Female	Reference	
		Male	1.30 (0.66,2.58)	0.451
	cT	cT1	Reference	
		cT2	1.73 (0.54,5.58)	0.361
		cT3	1.12 (0.39,3.18)	0.837
		cT4a	3.30 (0.70,15.61)	0.132
	cN	cN0	Reference	
		cN+	1.45 (0.66,3.19)	0.357
	Treatment	NACRT	Reference	
		NAC	0.12 (0.04,0.41)	0.001
RFS	Age	Continuous	1.01 (0.98,1.04)	0.448
	Sex	Female	Reference	
		Male	0.98 (0.54,1.75)	0.933
	cT	cT1	Reference	
		cT2	1.30 (0.48,3.48)	0.605
		cT3	1.03 (0.43,2.43)	0.950
		cT4a	1.86 (0.45,7.74)	0.395
	cN	cN0	Reference	
		cN+	1.13 (0.59,2.17)	0.718
	Treatment	NACRT	Reference	
		NAC	0.40 (0.20,0.84)	0.015

OS, overall survival; RFS, recurrence-free survival; NACRT, neoadjuvant chemoradiotherapy; NAC, neoadjuvant chemotherapy.

## Discussion

In this integrated analysis of IPD from phase III randomized trials, survival among patients with OSCC who achieved pCR differed significantly by neoadjuvant treatment modality. Although NACRT resulted in a higher pCR rate than NAC, patients who achieved pCR after NAC demonstrated significantly superior long-term survival compared with those who achieved pCR after NACRT. This finding corroborates the persistent discrepancy in prior trials in which improved pathological response with NACRT did not translate into superior OS. The results of the present study extend the observations of Cools-Lartigue *et al*.^18^, for oesophageal adenocarcinoma, to OSCC and indicate that the prognostic implications of pCR differ according to treatment modality. Collectively, the findings of the present study challenge the assumption that pCR represents an equivalent endpoint across regimens, while underscoring the need for modality-specific interpretation of pCR in neoadjuvant trials.

Based on this framework, several biological and clinical mechanisms may explain why patients who achieved pCR after NACRT experienced poorer survival than those who achieved pCR after NAC. First, although radiation provides strong locoregional control, its effect on micrometastatic disease may be limited. As a result, even when pCR is achieved at the primary site and regional lymph nodes, occult systemic disease may subsequently become evident after surgery. Recent advances in molecular residual disease (MRD) assessment using circulating tumour DNA (ctDNA) have shown that MRD status is strongly associated with the prognosis for resectable OSCC^33^  ^,34^. Therefore, incorporating MRD-based ctDNA analyses into comparisons of NAC *versus* NACRT could provide deeper insights into how the biological implications of pCR differ across treatment modalities. Second, chemoradiation-induced tumour regression may partly reflect radiation-induced cytotoxicity rather than inherent tumour chemosensitivity or immunogenicity^35^, resulting in a pCR phenotype that is less prognostically meaningful. Finally, in addition to these biological considerations, it is important to acknowledge that prior retrospective studies have faced substantial challenges in clarifying the relationship between pCR and long-term outcomes. Patients selected for NACRT often present with more locally advanced disease or less favourable baseline characteristics and such imbalances have made it difficult to determine whether the poorer post-pCR survival observed after NACRT reflects true biological differences or residual confounding related to tumour burden or host factors. In this regard, these methodological challenges were addressed in the present study by collecting individual patient data from multiple phase III RCTs and, importantly, restricting the analysis to patients who achieved pCR. Although this approach substantially improved comparability between treatment groups, the integration of heterogeneous RCTs, including those conducted in Eastern and Western populations and employing different chemotherapy regimens, meant that residual confounding could not be fully eliminated. Therefore, the findings should be interpreted with caution. Nevertheless, using an approach that was as methodologically strong as possible, given the available data, the design of this study facilitated the evaluation of survival differences in the post-pCR setting.

The appropriateness of using pCR as a surrogate endpoint for OS has also been repeatedly debated^36,37^. The authors of the present study previously demonstrated a lack of individual-level surrogacy between pCR and OS, suggesting that pCR may be unsuitable as a primary endpoint in phase III randomized trials^17^. However, this lack of individual-level surrogacy does not diminish the established role of pCR as a prognostic factor. Numerous studies have shown a strong association between pCR and OS in oesophageal cancer, supporting its value for prognostic stratification^38^. Importantly, correlation with OS is distinct from true surrogate validity. A lower HR for OS among patients who achieve pCR does not indicate individual-level surrogacy, which requires substantially stronger concordance, such as a rank correlation coefficient ≥0.8. Although such a criterion limits the use of pCR as a primary endpoint in phase III trials, an HR <1.00 remains sufficient for prognostic assessment and for guiding organ-preserving strategies. Accordingly, pCR may be appropriate as a primary endpoint in exploratory phase II trials but is unlikely to be suitable for phase III trials designed to accurately estimate OS benefit.

Taken together, the findings of the present study have important clinical implications for the management of locally advanced, resectable OSCC. Although NACRT achieves higher pCR rates, patients who achieve pCR after NAC demonstrate substantially superior long-term survival, supporting NAC as a key component of neoadjuvant treatment strategies. The results also suggest that patients who achieve clinical complete response (cCR) after NAC may have uniquely favourable tumour biology. As efforts to improve preoperative identification of cCR using imaging, endoscopy, biomarkers, and emerging approaches such as ctDNA-based MRD assessment continue to advance^39^, this population may represent an optimal candidate group for organ-preserving approaches, including surgery-avoidance and watch-and-wait strategies. Furthermore, emerging evidence indicates that neoadjuvant ICI-based therapy has begun to result in promising pCR rates among patients with OSCC, suggesting that the number of patients achieving pCR after neoadjuvant ICI-based therapy is likely to increase in the future^40^. In the present study, analysis of the causes of death revealed that no cancer-related deaths occurred among patients who achieved pCR after NAC, despite a non-negligible number of recurrences. This observation raises the hypothesis that patients who achieve pCR after NAC may have a broader range of effective salvage treatment options after recurrence, including radiotherapy and other additional therapies. Consistent with this hypothesis, a previous study showed that postoperative recurrence among patients with OSCC who responded to NAC frequently presented as a solitary lesion confined to the regional field without distant metastasis^41^. Suggests a higher likelihood of successful salvage treatment after NAC. Prospective studies are needed to evaluate the safety and oncological validity of treatment de-escalation strategies^42^. Furthermore, among patients managed with a non-surgical approach, those who achieve pCR after NACRT may require closer surveillance; however, the present findings reflect a relative comparison with pCR after NAC and the results of ongoing trials are needed to further clarify this issue^43^.

The present study has several limitations. First, data on recurrence patterns and details of post-recurrence treatment were unavailable. Patients who achieve pCR after NACRT are expected to have a lower incidence of locoregional recurrence than those treated with NAC^16,44^, which could help explain the observed discrepancy between pCR and OS; however, this could not be evaluated. Second, because the cohort included patients from both Asia and Europe, population heterogeneity and residual confounding may not have been fully eliminated. Additionally, restricting the analysis to patients who achieved pCR limited the sample size and precluded the inclusion of certain key covariates, most notably the chemotherapy regimen, in multivariable models. For these reasons, this study was designed not to compare relative efficacy between NAC and NACRT but rather to assess differences in post-pCR survival between the two treatment modalities. Third, among patients who achieved pCR after NAC in particular, the numbers of patients, death events, and recurrence events were very limited, resulting in wide confidence intervals for the Kaplan–Meier estimates. Thus, the results should be cautiously interpreted. Fourth, survival analyses from the time of pCR assessment could not be performed because only the date of randomization was available and potential immortal time bias was likely present. Finally, a central pathological review was not performed for ypT and ypN assessment, as this analysis was based on IPD collected from previously completed RCTs.

Although NACRT was associated with higher pCR and R0 resection rates, NAC was associated with significantly superior long-term survival for patients with resectable OSCC. The findings of the present study indicate that NAC should remain a central component of neoadjuvant treatment for locally advanced OSCC. Furthermore, if accurate preoperative identification of cCR after NAC becomes feasible, patients who achieve cCR after NAC may represent an optimal candidate group for future surgery-avoidance and watch-and-wait strategies.

## Supplementary Material

znag012_Supplementary_Data

## Data Availability

This study involved an integrated analysis of IPD collected from RCTs. The principal institution, Keio University, obtained approval from its Institutional Review Board. Keio University established data-sharing agreements with each participating institution and received data accordingly. Therefore, the data from this study cannot be shared with third parties, even upon request.
