# Supplementary material for: Survival after pathological complete response following neoadjuvant chemotherapy versus chemoradiotherapy for oesophageal squamous cell carcinoma
Source: Br J Surg. 2026 Feb 17;113(3):znag012. doi: 10.1093/bjs/znag012 (PMC13017761; doi:10.1093/bjs/znag012)
Supplement: znag012_Supplementary_Data [file znag012_supplementary_data.docx]

**Title:** Survival After Pathological Complete Response Following Neoadjuvant Chemotherapy vs Chemoradiotherapy in Esophageal Squamous Cell Carcinoma

Authors: Jun Okui^1,2^, Kengo Nagashima^3^, Satoru Matsuda^1^, Yasunori Sato^2^, Hirofumi Kawakubo^1^, Thomas Ruhstaller^4,5^, Peter Thuss-Patience^6^, Magnus Nilsson^7^, Fredrik Klevebro^7^, Lijie Tan^8^, Shaoyuan Zhang^8^, Thomas Aparicio^9^, Guillaume Piessen^10^, Charlène van der Zijden^11^, Bianca Mostert^12^, Bas P.L. Wijnhoven^11^, Takahiro Tsushima^13^, Hiroya Takeuchi^14^, Ken Kato^15^, and Yuko Kitagawa^1^

1. Department of Surgery, Keio University School of Medicine, Tokyo, Japan
2. Department of Biostatistics, Keio University School of Medicine, Tokyo, Japan
3. Biostatistics Unit, Clinical and Translational Research Center, Keio University Hospital, Tokyo, Japan
4. Swiss Group for Clinical Cancer Research (SAKK), Switzerland
5. University of Basel, Basel, Switzerland
6. Department of Hematology, Oncology and Cancer Immunology, Charité-University Medicine Berlin, Campus Virchow Klinikum, Berlin, Germany
7. Division of Surgery and Oncology, Department of Clinical Science, Intervention and Technology (CLINTEC), Karolinska Institutet and Department of Upper Abdominal Diseases, Karolinska University Hospital, Stockholm, Sweden
8. Department of Thoracic Surgery, Zhongshan Hospital, Fudan University, Shanghai, China
9. Department of Digestive Oncology, Hôpital Saint-Louis, APHP, Université Paris Cité, Paris, France
10. University Lille, CNRS, Inserm, CHU Lille, UMR9020-U1277 - CANTHER - Cancer Heterogeneity, Plasticity and Resistance to Therapies, F-59000, Lille, France
11. Department of Surgery, Erasmus MC Cancer Institute, Erasmus University Medical Center, Rotterdam, The Netherlands
12. Department of Medical Oncology, Erasmus MC Cancer Institute, Erasmus University Medical Center, Rotterdam, The Netherlands
13. Division of Gastrointestinal Oncology, Shizuoka Cancer Center, Shizuoka, Japan
14. Department of Surgery, Hamamatsu University School of Medicine, Shizuoka, Japan
15. Department of Head and Neck, Esophageal Medical Oncology, National Cancer Center  Hospital, Tokyo, Japan

***Corresponding Author**: Satoru Matsuda

Department of Surgery, Keio University School of Medicine, 35 Shinanomachi, Shinjuku-ku, Tokyo, 160-8582, Japan

Email: s.matsuda.a8@keio.jp

Phone: +81-3-5363-3802, Fax: +81-3-3355-4707

**Supplementary Materials - Index**

| **Supplementary Figures and Tables** |  |
| --- | --- |
| Figure S1. Patient flow diagram | *page 3* |
| Figure S2. Kaplan–Meier estimates of overall and recurrence-free survival in the non-pCR cohort | *page 4* |
| Table S1. Eligible studies identified by systematic review | *page 5* |

# Figure S1. Patient flow diagram.

Abbreviations: EC, esophageal cancer; EGJ, esophagogastric junction; ICI, immune checkpoint inhibitor; IPD, individual patient data; pCR, pathological complete response.


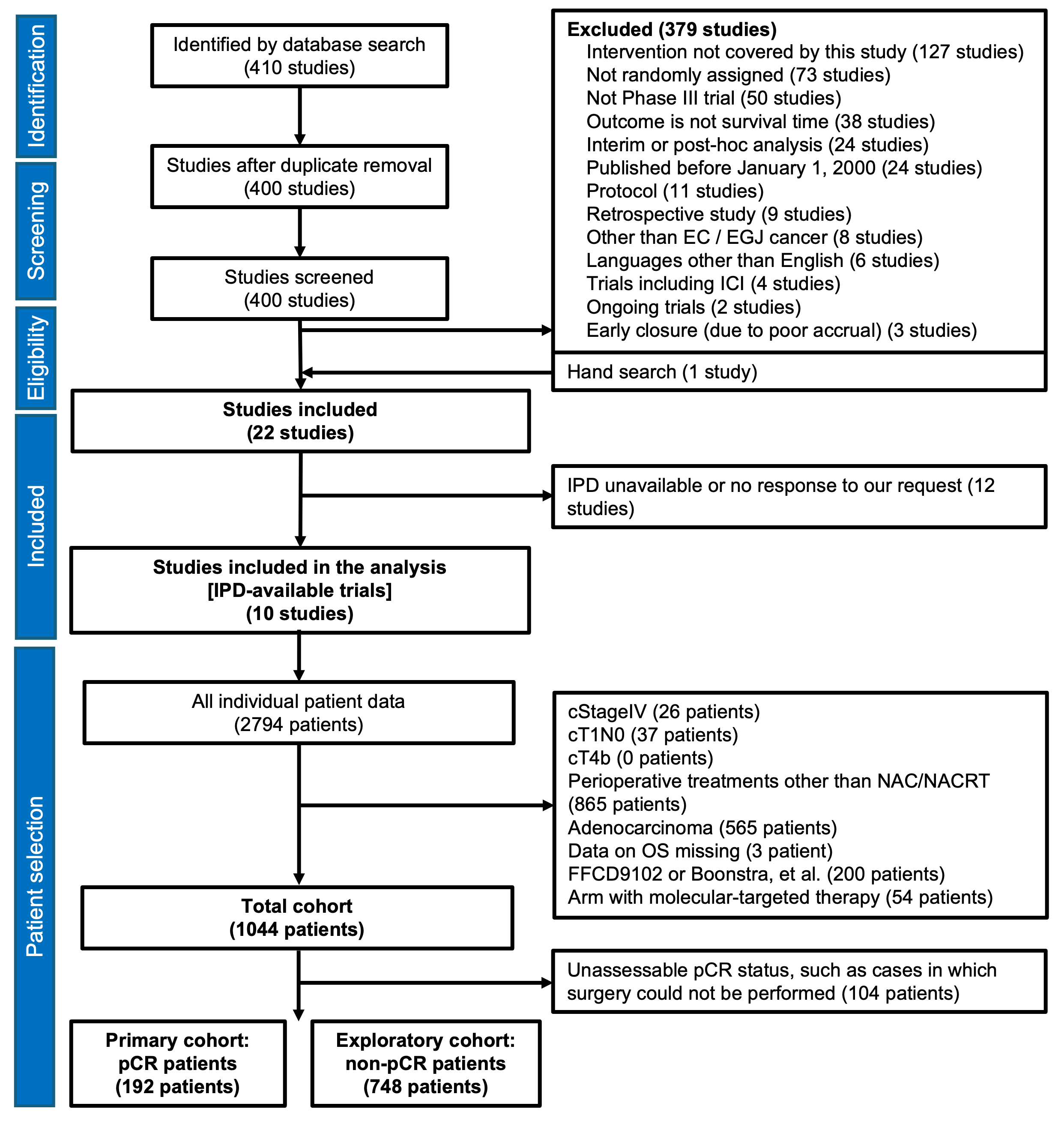


# Figure S2. Kaplan–Meier estimates of overall and recurrence-free survival in the non-pCR cohort.

Note: Shaded areas represent 95% confidence intervals. Abbreviations: OS, overall survival; pCR, pathological complete response; RFS, recurrence-free survival; SCC, squamous cell carcinoma.


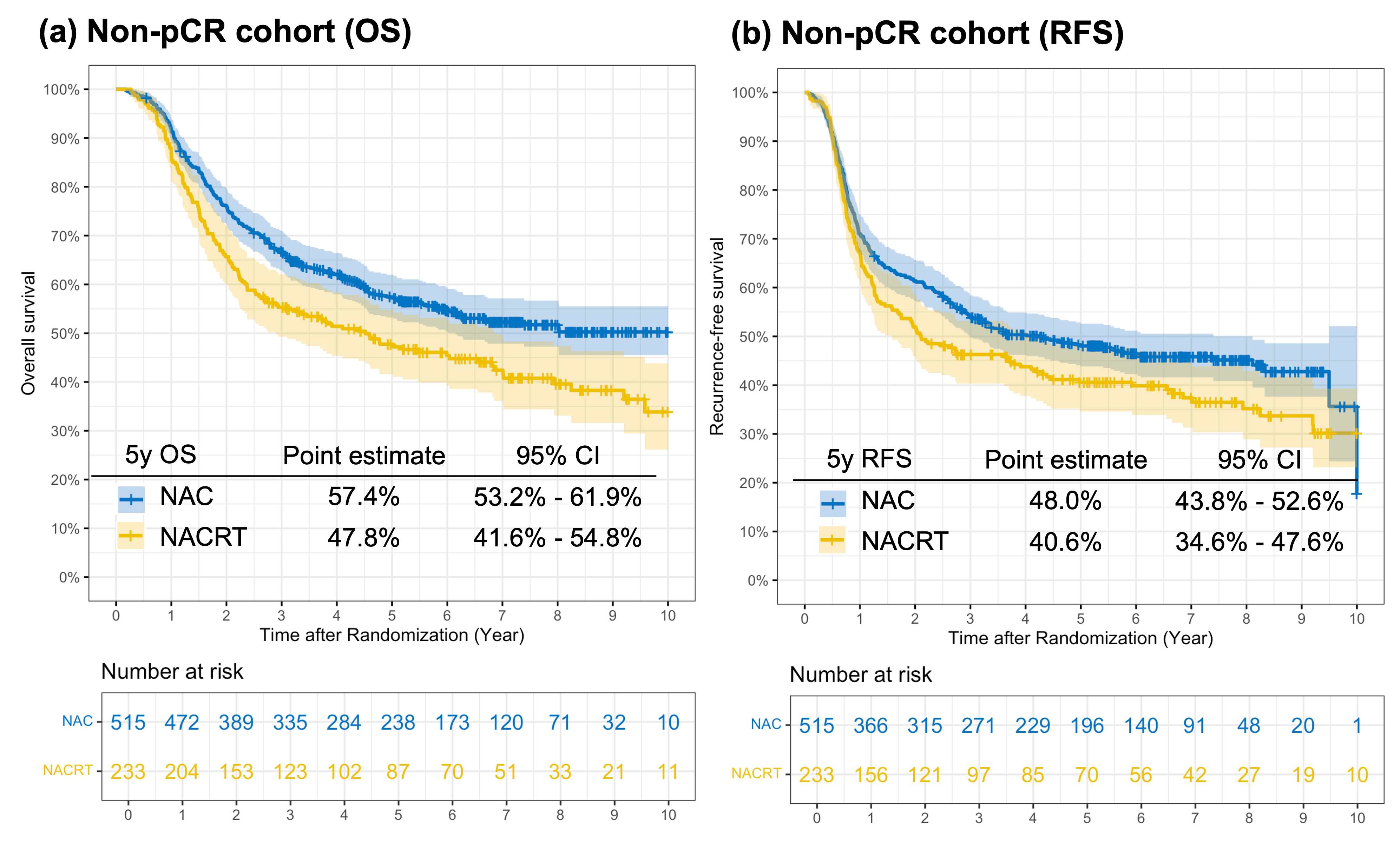


# Table S1. Eligible studies identified by systematic review.

| **Trial** | **Author** | **Published** | **Modality** | **RT** | **IPD** | **n** | **GEJC** | **SCC** | **AC** |
| --- | --- | --- | --- | --- | --- | --- | --- | --- | --- |
| JCOG1109* | Kato, et al. | 2024 | CS vs. CS | No | **Yes** | 401 | No | Yes | No |
| JCOG1109* | Kato, et al. | 2024 | CS vs. CRS | Yes | **Yes** | 399 | No | Yes | No |
| NeoRes2 | Nilsson, et al. | 2023 | CRS vs. CRS | Yes | **Yes** | 249 | Yes | Yes | Yes |
| Neo-AEGIS | ReyNolds, et al. | 2023 | CS vs. CRS | Yes | No | 362 | Yes | No | Yes |
| CMISG1701 | Tang, et al. | 2022 | CS vs. CRS | Yes | **Yes** | 264 | No | Yes | No |
| FLOT4 | Al-Batran, et al. | 2019 | CSC vs. CSC | No | No | 716 | Yes | No | Yes |
| SAKK75/08 | Ruhstaller, et al. | 2018 | CRS vs. CRS | Yes | **Yes** | 300 | Yes | Yes | Yes |
| NEOCRTEC5010 | Yang, et al. | 2018 | S vs. CRS | Yes | No | 451 | No | Yes | No |
| ST03 | Cunningham, et al. | 2017 | CSC vs. CSC | No | No | 1063 | Yes | No | Yes |
| OE05 | Alderson, et al. | 2017 | CS vs. CS | No | No | 897 | Yes | No | Yes |
| Zhao, et al. | Zhao, et al. | 2015 | CS vs. CSC | No | No | 346 | No | Yes | No |
| FFCD9901 | Mariette, et al. | 2014 | S vs. CRS | Yes | **Yes** | 195 | No | Yes | Yes |
| JCOG9907 | Ando, et al. | 2012 | CS vs. SC | No | **Yes** | 330 | No | Yes | No |
| CROSS | van Hagen, et al. | 2012 | S vs. CRS | Yes | **Yes** | 366 | Yes | Yes | Yes |
| Boonstra, et al. | Boonstra, et al. | 2011 | S vs. CS | No | **Yes** | 169 | No | Yes | No |
| FFCD9703 | Ychou, et al. | 2011 | S vs. CS | No | No | 224 | Yes | No | Yes |
| OE02 | Allum, et al. | 2009 | S vs. CS | No | No | 802 | No | Yes | Yes |
| FFCD9102 | Bedenne, et al. | 2007 | CRS vs. CRS | Yes | **Yes** | 259 | No | Yes | Yes |
| MAGIC | Cunningham, et al. | 2006 | S vs. CSC | No | No | 503 | Yes | No | Yes |
| Burmeister, et al. | Burmeister, et al. | 2005 | S vs. CRS | Yes | No | 256 | No | Yes | Yes |
| Lee, et al. | Lee, et al. | 2004 | S vs. CS | No | No | 101 | No | Yes | No |
| JCOG9204 | Ando, et al. | 2003 | S vs. SC | No | **Yes** | 242 | No | Yes | No |
| MRC | MRC | 2002 | S vs. CS | No | No | 802 | No | Yes | Yes |

**Abbreviations**: AC, adenocarcinoma; RT, radiotherapy; SCC, squamous cell carcinoma.

**Abbreviations of “Modality”**: C, Chemotherapy; R, radiotherapy; S, surgery.

CS, Neoadjuvant chemotherapy followed by surgery; CRS, Neoadjuvant chemoradiotherapy followed by surgery; S, Surgery alone; SC, Surgery followed by adjuvant chemotherapy.

***Note**: JCOG1109 included three arms (one control arm and two test arms), and therefore it is presented in two rows in the table.
